# Zika virus surveillance post‐epidemic in blood donors from São Paulo, Brazil 2016–2020

**DOI:** 10.1111/tme.70030

**Published:** 2025-10-16

**Authors:** Suzete Cleusa Ferreira, José Eduardo Levi, Anna Shoko Nishiya, Cesar de Almeida‐Neto, Jerenice Esdras Ferreira, Juliana Derriga, Nanci A. Salles, Silvia Petrossi Gallo Polato, Katia C. Dantas, Edmir Boturão‐Neto, Martha Mathias Rocha, Vanderson Rocha, Alfredo Mendrone‐Jr

**Affiliations:** ^1^ Departamento de Segurança Transfusional Fundação Pro Sangue Hemocentro de São Paulo, Divisão de Pesquisa São Paulo SP Brazil; ^2^ Laboratory of Medical Investigation in Pathogenesis and targeted therapy in Onco‐Immuno‐Hematology (LIM‐31), Department of Hematology, Hospital das Clínicas HCFMUSP, Faculdade de Medicina Universidade de São Paulo São Paulo SP Brazil; ^3^ Departamento de Virologia Universidade de São Paulo ‐Instituto de Medicina Tropical São Paulo SP Brazil; ^4^ Faculdade de Medicina da Universidade de São Paulo Disciplina de Ciências Médicas São Paulo SP Brazil; ^5^ Departamento de Hematologia e Bioquímica Instituto Adolf Lutz, Divisão de Patologia São Paulo SP Brazil; ^6^ Departamento de Patologia Faculdade de Medicina da Universidade de São Paulo São Paulo SP Brazil; ^7^ Departamento de Banco de Sangue Santa Casa da Misericordia de Santos São Paulo Brazil

**Keywords:** Zika virus, nucleic acid testing, blood donors, surveillance‐molecular biology

## Abstract

**Introduction:**

Zika virus (ZIKV) is primarily transmitted through the bite of the *Aedes aegypti* mosquito, though transmission via blood transfusion has also been documented. During the 2015 ZIKV epidemic in Brazil, severe complications were observed in pregnant women, leading to fetal microcephaly. This study evaluated the persistence of ZIKV in blood donated by healthy individuals during the post‐epidemic period from 2016 to 2020.

**Methods:**

Blood donor samples from 109 296 individuals were screened for ZIKV RNA using nucleic acids extracted from plasma pools (six donors per pool). These samples had previously undergone routine nucleic acid testing (NAT) for HBV, HCV and HIV.

**Results:**

Viral RNA (Ribonucleic Acid) was detected in a donor sample from the city of Santos in May 2016, resulting in a prevalence of 0.0009%. The positive donor was confirmed through viral sequencing using the Sanger method. Sequencing and phylogenetic analysis of an envelope gene amplicon revealed that the Zika virus RNA detected belonged to the Asian clade. This Asian lineage strain emerged in Brazil, Fortaleza, in 2015, isolated in northeastern Brazil in 2015, an area where most cases of microcephaly associated with ZIKV have been reported. A follow‐up sample collected 1 month after donation showed seroconversion.

**Conclusion:**

The detection of ZIKV RNA by NAT in a donated blood sample demonstrates that, although extremely rare, the virus is still present. Periodic active surveillance of blood donations for viruses associated with past outbreaks may help identify an incipient resurgence before it develops into a new epidemic.

## 1 INTRODUCTION

Brazil is a hotspot for emergent arboviruses, both autochthonous and introduced. Its tropical location and large urban centres with high population densities, infested by mosquitoes of the Culex and Aedes genera, offer an optimal environment for the proliferation of the vectors and consequently cause periodic outbreaks of arbodiseases.^1^

After its first isolation in 1947 from a sentinel Rhesus monkey in the Zika Forest in Uganda, Zika Virus (ZIKV) remained an obscure and rare infection among humans. Sixty years later, in 2007, a large outbreak was recorded in Yap Island in Micronesia.^2^ Subsequently, in 2013, a huge ZIKV outbreak occurred in French Polynesia.^3^ The virus crossed the Pacific Ocean and reached Brazil around 2013/2014.^2^, ^3^ First cases were detected in Brazil in early 2015, and microcephalic babies born from Zika‐infected women during pregnancy started to be observed in August 2015.^4^, ^5^, ^6^ When, in 2016, the causal link was evident, the World Health Organization (WHO) declared ZIKV a Public Health Emergency.^4^, ^5^, ^6^, ^7^

In May 2015, the city of Sumaré (121 km from the capital of São Paulo) recorded the first case of ZIKV in the State of São Paulo. A 52‐year‐old symptomatic man was initially diagnosed with a Dengue virus infection since both diseases have similar characteristics and Sumaré was facing a severe dengue epidemic, with 9208 confirmed cases. Curiously, ZIKV was only discovered in this individual because he had donated blood a few days before symptom initiation, and he provided post‐donation information of his dengue‐like illness. Units of his donated blood were transfused, comprising one of the few documented cases of transfusion transmitted (TT)‐ZIKV.^8^ The recipient of his platelet unit did not show any disease symptoms, while his fresh‐frozen plasma and red blood cells were not transfused.^7^, ^8^

West Nile Virus, another arthropod‐borne flavivirus, was also found to be TT and associated with major morbidity to recipients.^9^ This led to a concern that ZIKV represented another threat to blood supply safety. The WHO and blood transfusion societies adopted the precautionary principle of recommending the screening of donated blood for ZIKV RNA in affected areas, at least for units to be transfused into pregnant women and other high‐risk groups.^9^, ^10^

Fundação Pró‐Sangue Hemocentro de São Paulo (FPS) is a large blood centre that supplies blood to major public hospitals in São Paulo, such as the Hospital das Clínicas da Faculdade de Medicina de São Paulo, the largest public hospital complex in Latin America and a tertiary care centre. FPS receives an average of 150 000 blood donor volunteers per year. From these, ~115 000 units of whole blood and apheresis blood components are collected, resulting in the production of 290 000 blood components per year. The donor profile consists of 40% repeat donors, 30% first‐time donors, and 30% sporadic donors. Regarding gender, 40% are female, and 60% are male. In addition, 60% fall within the age range of 18–39 years. The Santa Casa de Santos is a small coastal blood bank that receives 14 276 donors per year, with 57.6% being men and 42.4% women. Donations are routinely submitted to mandatory screening for (HBV) Hepatitis B Virus (anti‐HBc/HBsAg/NAT), (HCV) Hepatitis C Virus (anti‐HCV/NAT), (HIV) Human Immunodeficiency Virus (Anti‐HIV + Ag/NAT), (HTLV 1 and 2) Antibodies against Human T‐Lymphotropic Virus types 1 and 2; anti‐Treponema and anti‐T. cruzi antibody testings are also performed. Nucleic acid testing (NAT) became mandatory in Brazil for HCV‐HIV in 2012 and for HBV in 2016. In September 2015, FPS decided to implement ZIKV‐NAT in a small number of units dedicated to pregnant women who needed blood transfusions. We decided to conduct ZIKV screening for a period of time. This programme remained in place until January 2016, when it was discontinued. Nevertheless, ZIKV‐NAT continued to be used for surveillance purposes. In the present manuscript, we describe the FPS experience over 4 years of ZIKV‐NAT post‐epidemic testing of blood donations.

## 2 METHODS

### 2.1 Clinical study design and sample collection

During the predonation interviews, several questions related to the possible viral or bacterial infection are asked, such as: have you had a fever, headaches or a skin rash in the last 15 days? Additionally, at the time of the study, we also asked during the pre‐donation interview whether the donor had been in a Zika virus endemic area.

The ZIKV‐NAT was performed from February 2016 to January 2020 under an investigational study protocol. NAT for HBV/HCV/HIV is routinely performed in plasma pools of six donations. Once per week, two 96‐well plates containing residual nucleic acid extracts from the plasma pools were used for the ZIKV‐NAT. Each 96‐well plate comprises 552 donations (552 = 6 × 92 pools + 4 controls). In total, 109 296 donations were evaluated by ZIKV‐NAT, 102 200 of which were blood donors from the FPS and 7096 from the Santos Blood Bank. In pools that showed reactivity for ZIKV, samples were tested individually.

### 2.2 Ethical approval

Human subjects research was conducted following approval from the Institutional Review Board (CAPPesq) of Hospital das Clínicas, University of São Paulo (online registration CAAE# 53233616.1.0000.065).

All blood donors provided written informed consent before sample collection, as required by the research ethics committee. For donors aged 16 or 17, signed consent was obtained from a parent or legal representative.

### 2.3 Zikv‐NAT

#### 2.3.1 Extraction of nucleic acids

Viral RNA from the blood donor sample was extracted from plasma pools comprising six donations, using the automated MDx system with the NAT HIV/HCV/HBV kit (Bio‐Manguinhos, Brazil). For pool preparation, 100 μL of plasma from each donation was combined, totaling 600 μL. From this volume, 267 μL were processed by the equipment for extraction, according to the manufacturer's instructions. Total nucleic acids were eluted in a final volume of 50 μL. After extraction, the samples were immediately stored at −80°C for a maximum of 48 h. They were then thawed and 10 μL of the RNA extract was used to perform the real‐time PCR test for ZIKV.

For each individual sample, RNA was extracted from 500 μL of serum or plasma using the MagNa Pure Compact Nucleic Acid Isolation—Large Volume kit (Roche, Germany) in an automated MagNa Pure Compact Instrument (Roche, Germany) and final elution in 100 μL from which 10 μL was directly used for ZIKV real‐time PCR.

#### 2.3.2 Real time PCR

Real‐time PCR was performed using a fast‐virus MasterMix (Thermofisher) and the primers and probe ZIKV‐F‐CCGCTGCCCAACACAAG, ZIKV‐R‐CCACTAACGTTCTTTTGCAGACAT, ZIKV‐PROBE‐FAM‐AGCCTACCTTGACAAGCAGTCAGACACTCAA, as described by Lanciotti et al.^11^ The reaction was performed using the StepOne System equipment (Applied Biosystems, Foster City, CA, USA), and the cycling conditions were 10 min at 95°C, followed by 45–50 cycles of 15 s at 94°C and 60 s at 60°C.

The limit of detection (LOD) with a 95% confidence interval (95% CI) was estimated at 7.5 copies/mL.

#### 2.3.3 Statistical Estimation of the LOD with 95% Confidence Interval

The LOD and its 95% confidence interval (95% CI) were estimated using a binary logistic regression model, in accordance with the guidelines provided by the Clinical and Laboratory Standards Institute (CLSI) EP17‐A2 (2012) [1]. Serial dilutions of known target concentrations (expressed in copies/mL) were prepared, and 20 replicate reactions were tested at each concentration level. The proportion of positive results at each dilution was used to model the probability of detection as a function of analyte concentration.

The logistic regression equation applied was:

$$\begin{array}{rcl} \text{logit}\left(p\right) & = & \beta 0+\beta 1\times \log10\left(\text{concentration}+1\right)\backslash \text{text\{logit\}}\left(p\right)\\ & = & \backslash \text{beta}\_0+\backslash \text{beta}\_1\backslash \text{times}\backslash \log\_\left\{10\right\}\left(\backslash \text{text\{concentration\}}+1\right)\text{logit}\left(p\right)\\ & = & \text{β0}+\text{β1}\times \log10\left(\text{concentration}+1\right) \end{array}$$

From the fitted model, the LOD—defined as the concentration at which there is a 95% probability of detection—was determined using the inverse of the logistic function:

LOD95%=10log0.95/0.05−β0β1−1\textLOD_95\%=10^\left\frac\log0.95/0.05−\beta_0\beta_1\right−1LOD95%=10β1log0.95/0.05−β0−1

The 95% CI for the LOD was calculated using the delta method **or** profile likelihood estimation, as implemented in standard statistical software (e.g., R or Python's statsmodels), based on the standard errors of the estimated regression coefficients.^12^

This method allows for robust estimation of the detection capability and its uncertainty, and is widely accepted for molecular diagnostic validations.

### 2.4 Sequencing and phylogenetic analysis

All samples that tested positive by RT‐PCR were subjected to Sanger sequencing. The 998‐base pair region of the Env gene was amplified using previously described primers and PCR conditions.^11^ The PCR product was purified using the QIAquick kit (Qiagen, Hilden, Germany) and directly sequenced using the Big Dye Terminator Cycle Sequencing Ready Reaction mix (Applied Biosystems, Foster City, USA). The sequences were edited using the Sequencer programme (version 4.1.4, Gene Codes Corporation, Ann Arbor, MI, USA) and the ZKV clade analyses were performed using the ZIKA Virus Typing Tool 3.68 online (https://www.genomedetective.com/app/typingtool/zika/).^13^

### 2.5 Serology

Detection of anti‐ZIKV IgM and IgG was performed using a commercial ELISA kit (Anti‐Zika Virus ELISA, EUROIMMUN, Lübeck, Germany), according to manufacturer's instructions.

## 3 RESULTS

From February 2016 to January 2020, a total of 109 296 donors' samples from FPS and Santa Casa de Santos were tested in pools of six, with an average of 27 000 donors tested per year.

**FIGURE 1:**
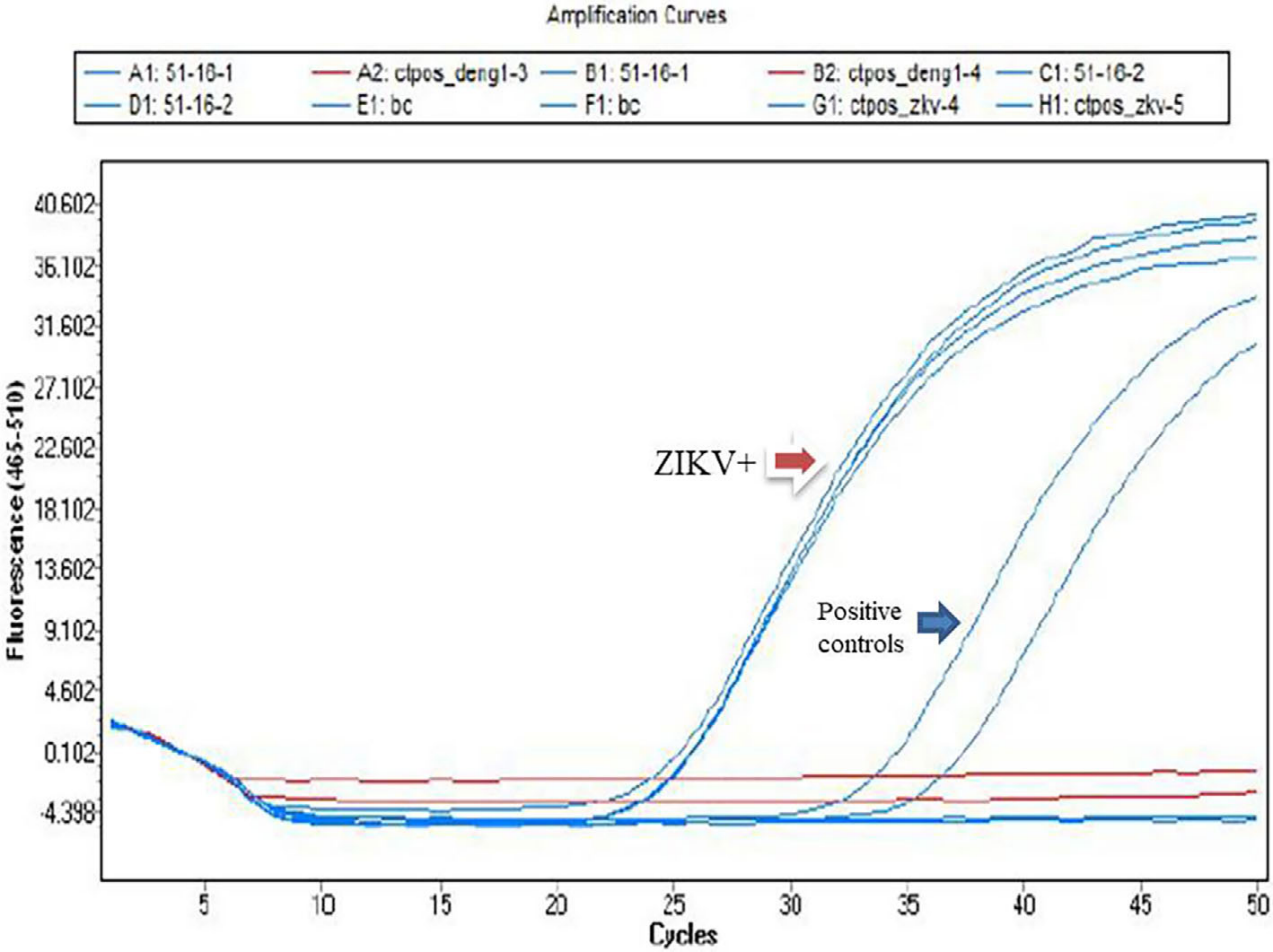
Real‐Time PCR reaction for ZIKV detection. The red arrow indicates the donor who tested positive.

**FIGURE 2:**
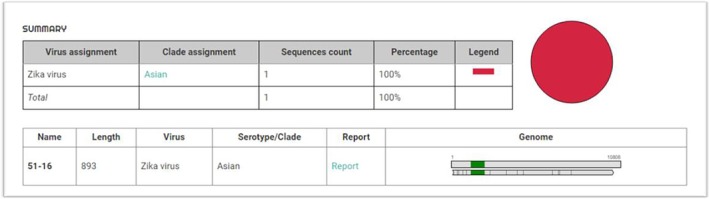
After Env‐gene sequencing and conducting an analysis using the Zika Virus Typing Tool on https://www.genomedetective.com/app/typingtool/zika/website, the Asian clade was determined for the donor who tested positive in the ZIKV NAT.

**FIGURE 3:**
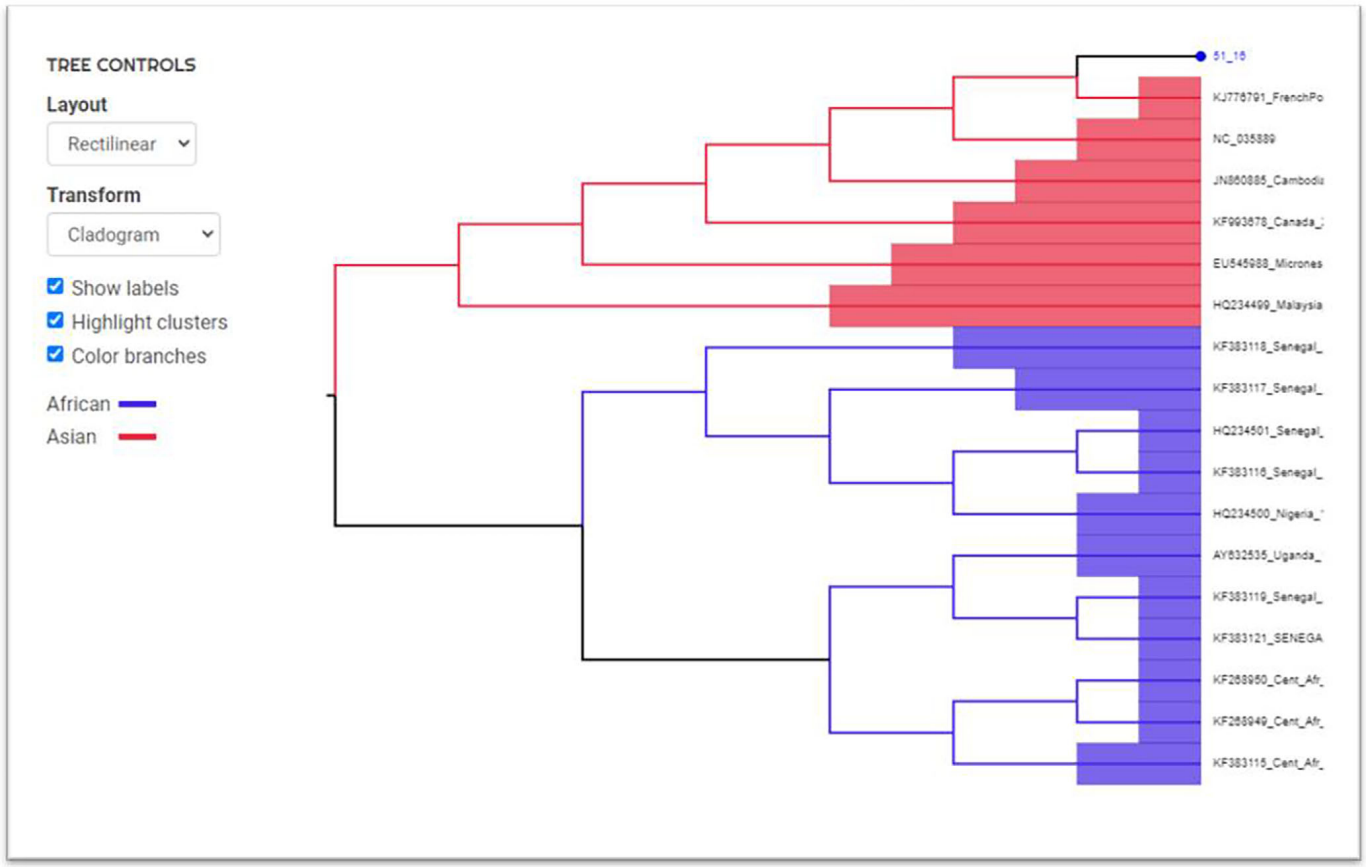
Phylogenetic analysis of the Env‐gene of ZIKV isolate from the donor who tested positive using the online Zika Virus Typing Tool at https://www.genomedetective.com/app/typingtool/zika/.

Viral RNA was detected in a donor sample from the city of Santos in May 2016, resulting in a prevalence of 0.0009%. The ZIKV pool nucleic acid test showed an amplification cycle threshold (Ct) value of 30.8, and in a subsequent individual test, the Ct was 24.7 (Figure 1). Phylogenetic analysis from an envelope gene amplicon showed it to be of the Asian Clade (Figures 2 and 3).

One month later, the donor was informed of the positive result for ZIKV. The collected bag was discarded, and a new sample was taken and submitted to serology in parallel to the index ZIKV‐RNA+ sample. The initial donation sample showed negative results for IgG and IgM, while the follow‐up sample collected 30 days later was reactive for both IgG and IgM. All samples from donors residing in the city of São Paulo were found non‐reactive for ZIKV‐RNA.

### 3.1 Statistical Estimation of the LOD

The LOD of the molecular test was estimated through statistical analysis based on binary logistic regression, following the recommendations of CLSI EP17‐A2 (2012). Samples with known target concentrations (in copies/mL) were used, with 20 replicates tested at each dilution level. The proportion of positive results per concentration was used to model the probability of detection (Table 1).

**TABLE 1:** Detection of the molecular target at different concentrations.

Concentration (copies/mL)	No. of replicates	No. of positives
1000	20	20
100	20	20
10	20	20
5	20	15
1	20	10
0	20	0

The analysis was conducted using the following logistic regression equation:

$$\text{logit}\left(p\right)=\beta ₀+\beta ₁\times \log10\left(\text{concentration}+1\right)$$

From the fitted model, the concentration corresponding to a 95% probability of detection was calculated using:

LOD=10^log0.95/0.05−β₀/β₁−1

**FIGURE 4:**
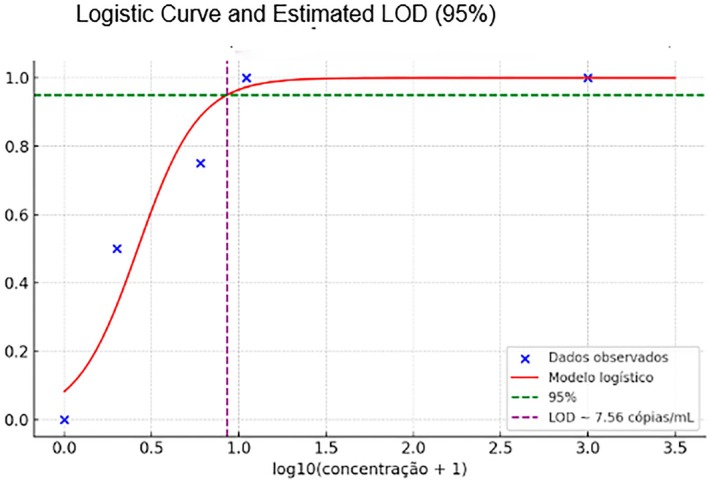
Logistic regression curve used to estimate the 95% limit of detection (LOD95%) based on the proportion of positive results per concentration level, following CLSI EP17‐A2 (2012).

The result obtained was: Statistical LOD = 7.56 copies/mL (Figure 4).

To evaluate whether the six‐sample pooling strategy impacted assay sensitivity, we compared Zika‐positive donor samples tested individually and in pools, using serial dilutions (1:2 to 1:64) in Zika‐negative serum. PCR analysis of both formats revealed no difference in the limit of detection. However, cycle threshold (Ct) values were consistently higher in the pooled samples, as shown in Table 2.

**TABLE 2:** Comparison of Ct values for individual and pooled Zika‐positive donor samples.

Dilution	Individual sample (Ct)	Pooled sample (Ct)
Undiluted	26	27
1/2	27	28
1/4	28	30
1/8	29	31
1/16	30	33
1/32	31	35
1/64	Not detected	Not detected

This comparison aimed to determine whether any Zika‐positive cases might have been missed due to the pooling approach. By performing parallel PCR on both individually diluted and pooled samples, we confirmed that the assay maintained its sensitivity across both testing formats. The slight increase in Ct values observed in pooled samples likely reflects the expected dilutional effect, but did not compromise the overall detection capability.

## 4 DISCUSSION

In view of the ZIKV epidemic throughout Brazil identified in 2015, a sensitive NAT methodology was established to ensure transfusion safety against this virus for specific recipient groups, that is, pregnant women. In 2016, only 10 cases were registered in São Paulo, followed by three in 2017 and no other cases from 2018 to 2023 were reported in the general populatio.^14^ Our finding of ZIKV viremic donations from 2016 to 2020 is consistent with the study by Lira et al. who did not identify any ZIKV donations among 3369 donations investigated by NAT from May 2016 to May 2018 in São Paulo, Capital.^15^ Similarly, Custer et al. 16 evaluated 994 370 donations between April 2016 and June 2019 in São Paulo, Belo Horizonte, Rio de Janeiro, and Recife and did not detect any ZIKV‐positive donations in 50 010 FPS donors. However, ZIKV‐positive donors were identified in other areas of the country during this period.^16^

We detected the presence of Zika virus RNA in a donor sample, which, after sequencing and phylogenetic analysis of an envelope gene amplicon, was identified as belonging to the Asian clade. This Asian lineage strain, first isolated in northeastern Brazil in 2015 (Brazil Fortaleza 2015; ZIKV/Brazil), emerged in Fortaleza city in the Northeast region, and is associated with most reported cases of ZIKV‐related microcephaly.^17^

Our detection of one viremic blood donation was from an individual residing in Santos. This coastal city, located 60 km from São Paulo, has consistently presented a higher incidence of Dengue when compared to São Paulo, and, thus, is considered to be a region with an elevated risk for infection by arboviruses.^18^

Our study findings reinforce that the difficult decision made by FPS to stop testing donated blood for the ZIKV was correct. It is extremely low prevalence did not justify the costs associated with continued routine testing. In the United States, ZIKV‐NAT was made mandatory in 2016, intercepting 383 viremic donations in this year, most from Puerto Rico. In the following year, 17 reactive donations were identified and in 2018 only 2 were positive. From 2019 to 2021 the positive ZIKV‐NAT yield on millions of blood samples was zero. This finally led the FDA to withdraw the ZIKV‐NAT recommendation.^19^

The recurrence of a Zika epidemic in Brazil cannot be predicted. Country‐wide there were approximately 8425 ZIKV‐positive cases in 2023.^20^ This is a small number when considering that the population of Brazil is 215 million. In this context, our blood centre will continue periodic selective screening of blood donations in the São Paulo region for ZIKV to monitor changes in its occurrence and, thereby, to mitigate the risk of Zika virus transmission and to ensure a safe blood supply.

## AUTHOR CONTRIBUTIONS

**Suzete Cleusa Ferreira:** Resources; project administration; methodology; investigation; conceptualization; formal analysis; visualisation; validation; writing—original draft; writing—review and editing. **José Eduardo Levi:** Data curation; funding acquisition; writing—original draft; writing—review and editing. **Anna Shoko Nishiya:** Investigation; methodology; validation; resources; writing—review and editing. **Cesar de Almeida‐Neto:** Formal analysis; writing—review and editing. **Jerenice Esdras Ferreira:** Resources; formal analysis; writing—reviewed and editing. **Juliana Derriga:** Resources; validation; methodology. **Nanci A. Salles:** Resources; methodology. **Silvia Petrossi Gallo Polato:** Visualisation. **Katia C. Dantas:** Formal analysis; writing—review and editing. **Edmir Boturão‐Neto:** Formal analysis; writing—review and editing. **Martha Mathias Rocha:** Formal analysis; writing—review and editing. **Vanderson Rocha:** Formal analysis; writing—review and editing. **Alfredo Mendrone‐Jr:** Project administration; supervision; writing original draft; writing—review and editing.

## FUNDING INFORMATION

This project was funded by the Fapesp (São Paulo Research Foundation) under grant number 2014/50093‐8.

## CONFLICT OF INTEREST STATEMENT

The authors have no competing interests.

## Data Availability

Data sharing does not apply to this article as all data is available in the results. The sequence generated by the sequencing has even been submitted to GenBank and the reference number is given in the text of the article.
